# Analysis of Genetic Variation in *Brevipalpus yothersi* (Acari: Tenuipalpidae) Populations from Four Species of Citrus Host Plants

**DOI:** 10.1371/journal.pone.0164552

**Published:** 2016-10-13

**Authors:** Delfina Salinas-Vargas, Ma. Teresa Santillán-Galicia, Ariel W. Guzmán-Franco, Antonio Hernández-López, Laura D. Ortega-Arenas, Gustavo Mora-Aguilera

**Affiliations:** 1 Posgrado en Fitosanidad-Entomología y Acarología, Colegio de Postgraduados, Texcoco, Edo. de Mexico, Mexico; 2 Escuela Nacional de Estudios Superiores, ENES, Unidad Leon (UNAM), Leon, Guanajuato, Mexico; 3 Posgrado en Fitosanidad-Fitopatología, Colegio de Postgraduados, Texcoco, Edo. de Mexico, Mexico; University of Arkansas, UNITED STATES

## Abstract

We studied species diversity and genetic variation among populations of *Brevipalpus* mites from four species of citrus host plants. We sampled mites on orange, lime, grapefruit and mandarin trees from orchards at six localities distributed in the five most important citrus producing states in Mexico. Genetic variation among citrus host plants and localities were assessed by analysis of nucleotide sequence data from fragments of the mitochondrial cytochrome oxidase subunit I (COI). Both *Brevipalpus yothersi* and *B*. *californicus* were found at these sites, and *B*. *yothersi* was the most abundant species found on all citrus species and in all localities sampled. *B*. *californicus* was found mainly on orange and mandarin and only in two of the states sampled. AMOVA and haplotype network analyses revealed no correlation between *B*. *yothersi* genetic population structure and geographical origin or citrus host plant species. Considering that a previous study reported greater genetic diversity in *B*. *yothersi* populations from Brazil than we observed in Mexico, we discuss the possibility that the Mexican populations may have originated in the southern region of America.

## Introduction

*Brevipalpus* Donnadieu is the most economically important mite genus within the family Tenuipalpidae [1]. *Brevipalpus* contains more than 300 species that have a worldwide distribution [2, 3]. *Brevipalpus* mites are commonly parthenogenetic (females producing females) and males are only rarely found in some species [4]. Most *Brevipalpus* species are economically important because they feed on agricultural crops and some of them also transmit viruses to host plants, including *Citrus sinensis* (L.) Osbeck [5–7], *Coffea arabica* L. (Gentianales: Rubiaceae) [8], *Passiflora edulis* Sims (Malpighiales: Passifloraceae) [9, 10] and some ornamental species in the genera *Angraecum*, *Diplocaulobium*, *Stanhopea*, *Miltonia*, *Hormidium* [11, 12], *Xylobium*, *Oncidium*, *Trichopilia* [13] and *Cymbidium* [14]. *Brevipalpus phoenicis s*.*l*. Geijskes, *Brevipalpus californicus* Banks, and *Brevipalpus obovatus* Donnadieu are considered amongst the most important agricultural pests and all are virus vectors [15, 16].

Beard et al. [17] described the existence of morphospecies within *B*. *phoenicis*; however, these morphospecies have been recently elevated to full species status [18]. Using molecular techniques, Sanchez-Velazquez et al. [19] successfully identified the existence of two of these newly-described species in Mexico: *Brevipalpus yothersi* (Baker), which was the most abundant species, and *Brevipalpus papayensis* (Baker). *Brevipalpus californicus* was also recorded but at very low densities. Even though 22 samples were collected in Brazil, a comparison of the population genetic structure of *B*. *yothersi* from Mexico and Brazil showed that 14 haplotypes were observed in Brazilain populations while only four haplotypes were identified in the 37 samples from Mexico [19]. A diversity of factors are thought to influence genetic variation amongst populations of mites including geographical distance and population density. Low population densities can lead to habitat fragmentation and limited gene flow [20–22]. However, adaptation to host plants by herbivorous arthropods can also play a major role in their differential genetic structure or even lead to speciation [23].

The main difference between samples from Mexico and Brazil was that the mites from Brazil were collected from five different varieties of orange, while all the samples from Mexico were collected from the same variety of orange [19]. We hypothesized that the greater genetic diversity found in Brazilian populations compared with Mexican populations was due to the diversity of host plants from which they were sampled. Based on this, and using a fragment of the mitochondrial gene cytochrome oxidase subunit I (COI), we determined the genetic population structure of *B*. *yothersi* collected from four different citrus species from five of the most important citrus-producing areas in Mexico.

## Materials and Methods

### Host plants and sample collection

Mites were collected from four different citrus species: sweet orange (*C*. *sinensis*), grapefruit (*Citrus paradisi* Macfad.), mandarin (*Citrus reticulata* Blanco) and lime (*Citrus latifolia* (Yu. Tanaka) Tanaka). Sample collections were made in the municipalities of Culiacan and Guasave in Sinaloa, Mugica in Michoacan, Montemorelos in Nuevo León, Oxkutzcab in Yucatan and Martinez de la Torre in Veracruz. The locations were selected because they all had orchards containing the four citrus species under evaluation, except Veracruz where no grapefruit could be found. The study was conducted in private orchards with the permission of the landowners. The field studies did not involve endangered or protected species.

The sampling methodology was similar for all citrus species. Mites were collected from both fruits and leaves from five trees with evident signs of mite feeding damage. In the field, and with the aid of a stereomicroscope, only the female adults of *Brevipalpus* species were collected for transportation to the laboratory. These female mites were inoculated on to fruits of the same species from the same orchard that had been prepared as described below. Each fruit was washed with soapy water, rinsed and allowed to dry for 12 hours. Half of each fruit was immersed in liquid wax which was allowed to solidify, and a thin line (approx. 2 mm width) of insect barrier glue (Agralan Ltd, Ashton Keynes. Wiltshire, UK) was placed around each orange, separating the wax-covered half from the clean half. Three lines (3 cm long × 2 mm width) made from a mixture of sand, flour and plaster in equal proportions were placed on the clean half of each orange (without wax) to provide refuges and oviposition sites for the mites. Approximately 10–50 adult female mites were collected and introduced on to each fruit. Once the mites had been introduced, each prepared fruit was then placed inside a plastic container (13 x 12 x 8 cm) on a layer of expanded polystyrene foam (2 cm thick). The wax-covered section of the fruit was embedded in the polystyrene layer to prevent the fruit from moving inside the container during transportation to the laboratory. Twenty-five fruits were prepared for each citrus × location combination resulting in a total of 575 fruits. No grapefruit could be found in Veracruz.

In the laboratory, one adult female was randomly selected from each fruit and placed on to a different fruit of the same citrus species that had been prepared as described previously. All fruits, each bearing a single female adult mite, were placed individually inside plastic containers and incubated at 25 ± 2°C, 60% RH and 12:12 light: dark regime. Fruits were replaced when necessary if dehydration was evident. Fruits were maintained under these conditions until the first generation of adult mites had developed.

### Species diversity and analysis of the effect of host plant species on the genetic population structure of *Brevipalpus* species

#### DNA extraction and PCR protocols

Genomic DNA was extracted from two first-generation adult mites per fruit, each representing an experimental sample. For this, the DNeasy Blood & Tissue (QIAGEN, Germantown, MD, USA) kit was used following the manufacturer’s instructions. A fragment of the gene COI was amplified using the primers DNF (TGA TTT TTT GGT CAC CCA GAA G) and DNR (TAC AGC TCC TAT AGA TAA AAC) [24]. PCR reactions were done in a 25 μL reaction volume containing 2.5 μL of buffer 10X (600 mM Tris-SO4 (pH 8.9), 180 mM ammonium sulphate), 1 mM of MgCl2, 0.2 μM of each primer, 0.2 mM of dNTP’s, 0.5 μL of Taq DNA polymerase (Quiagen, GmbH, Hilden, Germany) and 5 μL (approx. 20 ng) of DNA. PCR amplifications were done using a MyCycler (BIO-RAD Laboratories Inc., Hercules, CA, USA). Thermal conditions used were as follows: one cycle of 4 min at 94°C, followed by 35 cycles of 60 s at 94°C, 60 s at 54°C and 60 s at 72°C with a final extension at 72°C for 5 min. PCR products were visualized on 1% agarose gels in 1X TAE. GelPilot 100 bp Plus (QIAGEN, GmbH, Hilden, Germany) size markers were used. The gels were stained with ethidium bromide (0.1 μg mL^–1^) and photographed. All PCR products were sent to the company Macrogen Inc. (South Korea) for direct sequencing.

#### Data analysis

Sequence traces were edited and assembled using BioEdit v.7.1.9 [25]. Multiple alignments were made using Clustal W [26] implemented in BioEdit using the default alignment parameters set in the program. All sequences were analysed using maximum likelihood in Molecular Evolutionary Genetic Analysis (MEGA) ver. 6.0 for Windows, with the Close-Neighbour-Interchange algorithm [27]. The robustness of branches was estimated by bootstrap analysis with 1000 repeated samplings of the data [28]. Tree reconstruction was made excluding non-synonymous substitutions, without any effect on tree topology. We show the tree including all sites. For the taxonomic placement of the samples, additional sequences from different *Brevipalpus* species were retrieved from GenBank. The sequence from *Cenopalpus pulcher* (Canestrini and Fanzago) (Acari: Tenuipalpidae) was retrieved from GenBank and used as the outgroup for this analysis. In addition, the Nei-Gojobori method [29], as implemented in the Z test in the program MEGA 6.0 [27], was used to compute the synonymous and nonsynonymous distances at a 5% significance level.

Genetic differences amongst haplotypes within each *Brevipalpus* species sampled were detected in a maximum parsimony network [30] using TCS v. 1.21 [31]. The connection limit amongst haplotypes (limits of parsimony) was set to the default value of 95%, and where gaps were treated as a 5th state. For the following analyses, only the samples from *B*. *yothersi*, the most abundant species after the phylogenetic analysis (see results section), were used. The partition of genetic variation amongst host citrus species populations and amongst populations from different geographical origins were analysed separately, and each analysis was followed by a comparison amongst all populations by analysis of molecular variance (AMOVA), estimated by computing F-statistics using Arlequin v. 3.5 [32] with 10000 permutations. In addition, the Mantel test was conducted to assess the correlation between genetic and geographic distances using the Isolation by Distance (IBD) web service [33].

## Results

### Species diversity in the genus *Brevipalpus*

**Fig 1:**
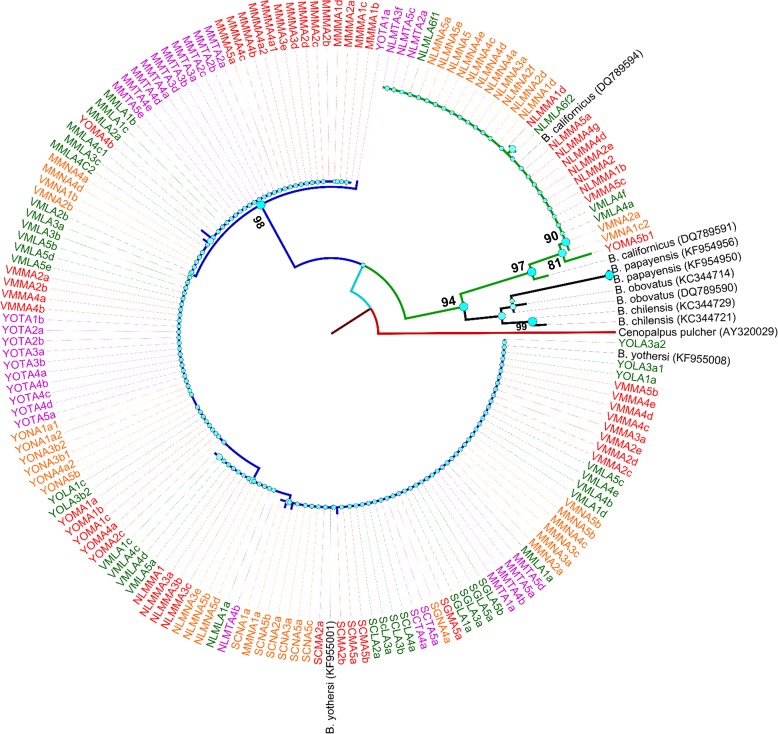
Dendogram inferred from maximum likelihood analysis of COI data from *B*. *yothersi* and *B*. *californicus*. The first letter (or two letters) in each sample name represents the Mexican state from which mite samples were collected: Sinaloa (S), Michoacan (M), Veracruz (V), Nuevo Leon (NL) and Yucatan (Y). Different colours represent different citrus species from which mite samples were collected: orange (**N)**, mandarin **(M)**, grapefruit **(T)** and lime **(L)**. Other *Brevipalpus* species used as reference species and *Cenopalpus pulcher* (Canestrini and Fanzago) (Acari: Tenuipalpidae) used as the outgroup, are labelled according to their GenBank accession numbers. Only bootstrap values above 90% were considered.

One hundred and sixty sequences were obtained in total. After alignment and trimming, all sequences were truncated to 362 bp for *B*. *yothersi* and to 427 bp for *B*. *californicus*. Sequences contained 329 non-variables sites, 98 variable sites and 53 sites with parsimonious information. The phylogenetic analysis showed the existence of only two species of *Brevipalpus* in the samples, *B*. *yothersi* (131 samples, S1 Table) and *B*. *californicus* (29 samples, S2 Table). These *Brevipalpus* species were clearly separated from each other by bootstrap values above 90% (Fig 1).

### Genetic variation amongst *B*. *yothersi* populations

**Fig 2:**
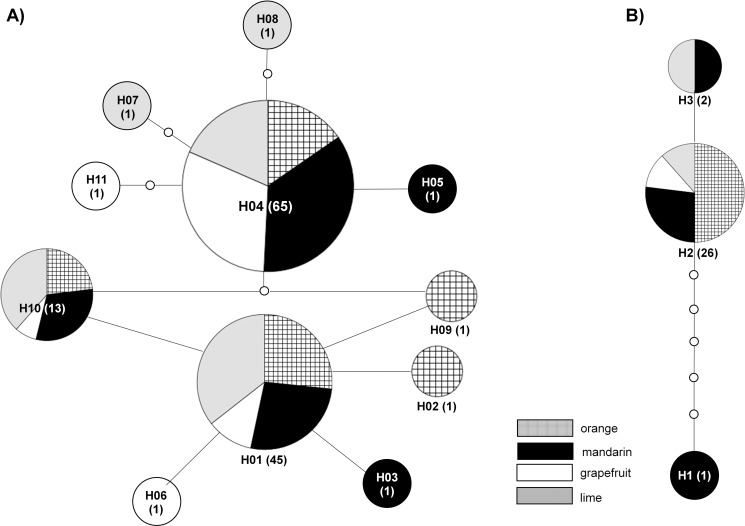
**Most parsimonious haplotype network for the 11 haplotypes found in *B*. *yothersi* (A) and the three haplotypes found in *B*. *californicus* (B).** Haplotypes are connected with a 95% confidence limit. Each line in the network represents a single mutational change. Small white circles indicate missing haplotypes. Numbers of samples per haplotype are shown in parentheses.

**Fig 3:**
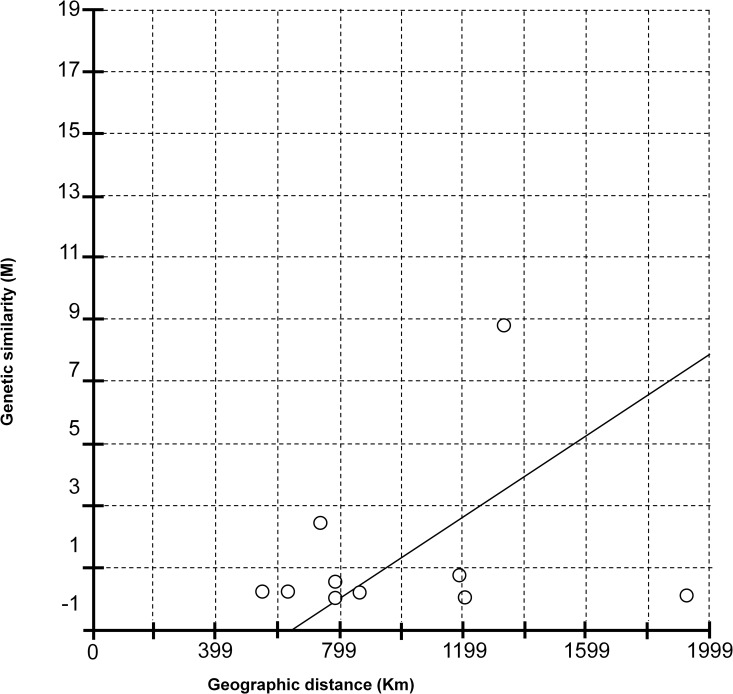
Mantel test graph. The graph shows the correlation between genetic variation (based on COI sequence data) and distance (Km) found in *B*. *yothersi* populations.

Haplotype network analysis revealed the existence of 11 haplotypes (Fig 2A). The most common haplotype was H04 with 65 samples of which ten were from orange, 23 from mandarin, 20 from grapefruit and 12 from lime. The second most abundant haplotype was H01 with 45 samples of which 12 were from orange, 12 from mandarin, 5 from grapefruit and 16 from lime. Haplotype H10 contained 13 samples, of which three were from orange, four from mandarin, one from grapefruit and five from lime. Haplotypes H02, H03, H05, H06, H07, H08 and H09 contained only one sample each. AMOVA analysis showed that only 5.9% of the variation could be explained by the host plant species from which the mites were collected, while 94.1% of the variation was explained by comparisons amongst samples regardless of the host plant species (Table 1). When the effect of geographical origin was analysed, 44.5% of the variation could be explained by geographical origin and 55.45% could be explained by the variation amongst populations regardless of geographical origin. Although the effect of geographical separation after AMOVA analysis showed a 44.55% contribution for the overall genetic variation, Mantel tests on pairwise genetic and geographic distances did not show a significant isolation by distance (IBD) pattern (*r* = 0.2141, *P* = 0.7810) (Fig 3).

**Table 1:** Results of the analyses of molecular variance (AMOVA) of COI sequences from *B*. *yothersi* collected from four different citrus species from five localities. All tests were based on both molecular distances and haplotype frequencies. Statistical significance: P<0.001 (**) and NS = not significant.

Source of variation	d.f.	Sum of squares	Variance components	Variation (%)
Amongst populations (citrus species)	3	7.13	0.049	5.89^NS^
Within populations	127	99.69	0.785	94.11**
Total	130	106.82	0.834	
Amongst populations (geographical origin)	4	42.87	0.408	44.55**
Within populations	126	63.95	0.508	55.45**
Total	130	106.82	0.916	

### Genetic variation amongst *B*. *californicus* populations

Haplotype network analysis revealed the existence of three haplotypes (Fig 2B). The most common haplotype was H2 containing 26 samples, of which 13 were from orange, seven from mandarin, three from grapefruit and three from lime. Haplotype H3 contained two samples, one from mandarin and one from lime, and haplotype H1 included one sample from mandarin.

## Discussion

Our results confirm the presence of *B*. *yothersi* in orchards from the five Mexican states where samples were collected. *Brevipalpus californicus* was only found in Nuevo Leon and Veracruz consistent with earlier observations [34, 19]. Our haplotype analysis revealed 11 haplotypes in *B*. *yothersi* populations. This represents a greater number of haplotypes compared with the study of Sanchez-Velazquez et al. [19] who only reported four haplotypes for Mexico. However, our results showed that there was no significant correlation between haplotype diversity and the citrus species from which the mites were collected. The different number of haplotypes reported by Sanchez-Velazquez et al. [19] compared to the present study could be related to the number of samples analysed, as those authors analysed 37 sequences obtained from *B*. *yothersi*, compared with our study where we used 131 sequences from the same mite species. Interestingly, limited samples of mites from Brazil (only 22 samples) still showed a greater genetic diversity, with 14 haplotypes, compared with either our current study or the study of Sanchez-Velazquez et al. [19] of Mexican samples. Furthermore, the AMOVA analysis showed insignificant variation in haplotype diversity due to host plant species, ruling out the potential existence of host associated differentiation (HAD) in *B*. *yothersi* populations.

HAD in herbivores can be found at different taxonomic levels of the host. For example, amongst individual plants of the same species [35], different populations within the same plant species [36] or amongst different plant species [37]. HAD has been reported previously for *B*. *phoenicis* s.l. mites collected from orange, hibiscus (*Hibiscus rosa-sinensis* L.) (Malvales: Malvaceae) and acerola (*Malpighia glabra* L.) (Malpighiales: Malpighiaceae); when populations from each of these hosts plants were transferred on to a different host plant species they failed to reproduce suggesting a close adaptation to the original host plant [38]. It is likely that HAD could still be detected in *B*. *yothersi* populations if mites had been collected from plant species from different genera or families rather than just different species within the same genus. For example, *B*. *yothersi* has been reported on other hosts such as coconut, *Cocos nucifera* L. (Arecales: Arecaceae); sunflower, *Helianthus annuus* L. (Asterales: Asteraceae) and avocado, *Persea americana* Mill. (Laurales: Lauraceae) [17], although not evaluated here these populations may be different from the populations on citrus.

It is possible that the Mexican *B*. *yothersi* populations could have originated from the Brazilian populations. It is assumed that, if greater genetic variation is found in a particular region, that this region could be considered as the potential place of origin, from where only a few haplotypes successfully adapt and migrate into other regions [39]. A preliminary haplotype analysis using Brazilian sequences from *B*. *yothersi* obtained by Sanchez-Velazquez et al. [19] (data not shown) and sequences obtained in our current study showed that from the 14 haplotypes obtained from the Brazilian samples, two were also found in our Mexican samples, partially confirming our hypothesis that the origin of the Mexican *B*. *yothersi* populations was Brazil. In addition, as reported by Groot et al. [38], *B*. *phoenicis* s.l. can be host specific, failing to reproduce on alternate hosts, which could lead to local extinction of haplotypes and reduced genetic variation.

We found more *B*. *yothersi* compared with *B*. *californicus*. This result was similar to previous reports for Mexico [34, 19] and suggests that *B*. *yothersi* could be more successful in colonizing different citrus species in different regions compared with *B*. *californicus*. *Brevipalpus yothersi* was collected from all citrus species and regions, whereas *B*. *californicus* was only found in two Mexican states (Nuevo Leon and Veracruz) and mainly on orange and mandarin. When we were rearing through the first generation adults for DNA extraction, most of the field *B*. *californicus* adults that were placed on individual fruits failed to reproduce, which significantly reduced the number of samples we could analyse. It is possible that *B*. *californicus* has another preferred host, which was not studied here, but certainly warrants further investigation.

The forces influencing observed levels of genetic diversity in *B*. *yothersi*, while unrelated to host plant use (Table 1), could be partially attributed to the different control strategies carried out in each region. Although the use of acaricides is the most common control method, the frequency and type of acaricide may vary amongst regions. Indeed, the effect of the control methods used against herbivore populations and its relationship with their genetic population structure has been reported previously. Populations of *Tetranychus urticae* Koch (Trombidiformes: Tetranychidae) in mandarin orchards showed higher genetic diversity when mites were collected in orchards under an integrated pest management (IPM) program compared with mites collected in orchards under organic management (OM) [40]. These authors suggest that the alternation of some acaricides used in the IPM orchards could be responsible for the different population genetics observed.

In conclusion, *B*. *yothersi* was found on all citrus species and in all regions sampled. *Brevipalpus californicus* were only found in two regions and mostly on orange and mandarin. Genetic variation in *B*. *yothersi* populations was not related to host plant use or geographical origin. Considering that greater genetic variation in *B*. *yothersi* populations from Brazil compared with Mexico has been reported previously, we suggest that the Mexican populations may have originated from this southern region of America.

## Supporting Information

S1 Table List of samples of *Brevipalpus yothersi*. GenBank accession numbers, locality and haplotype assignment after the haplotype network analysis are provided. List of sequences used as a reference for the genetic comparison are shown. SL = Sinaloa, MN = Michoacan, VZ = Veracruz, NL = Nuevo Leon, YN = Yucatan.
(DOC)

S2 Table List of samples of *Brevipalpus californicus* obtained from different host plant species. GenBank accession numbers, locality and haplotype assignment after the haplotype network analysis are provided. List of sequences used as a reference for the genetic comparison are shown. VZ = Veracruz, NL = Nuevo Leon, YN = Yucatan.
(DOC)
