# Supplementary material for: Analysis of Genetic Variation in Brevipalpus yothersi (Acari: Tenuipalpidae) Populations from Four Species of Citrus Host Plants
Source: PLoS One. 2016 Oct 13;11(10):e0164552. doi: 10.1371/journal.pone.0164552 (PMC5063392; doi:10.1371/journal.pone.0164552)
Supplement: S1 Table — GenBank accession numbers, locality and haplotype assignment after the haplotype network analysis are provided. List of sequences used as a reference for the genetic comparison are shown. SL = Sinaloa, MN = Michoacan, VZ = Veracruz, NL = Nuevo Leon, YN = Yucatan. (DOC) [file pone.0164552.s001.doc]

**S1 Table. List of samples of *Brevipalpus yothersi****.* GenBank accession numbers, locality and haplotype assignment after the haplotype network analysis are provided. List of sequences used as a reference for the genetic comparison are shown. SL= Sinaloa, MN= Michoacan, VZ=Veracruz, NL= Nuevo Leon, YN=Yucatan.

| Location | Sample | Host | Haplotype | GenBank | Geographical coordinates (North, West) |
| --- | --- | --- | --- | --- | --- |
| *Brevipalpus yothersi* | | | | | |
| Culiacan, SL | SCNA1a | orange | H01 | KX100162 | 24° 54.903, 107° 34.166 |
|  | SCNA2a | orange | H01 | KX100163 | 24° 54.863, 107° 34.180 |
|  | SCNA3a | orange | H01 | KX100164 | 24° 54.845, 107° 34.168 |
|  | SCNA5a | orange | H01 | KX100165 | 24° 54.811, 107° 34.159 |
|  | SCNA5b | orange | H02 | KX100166 | 24° 54.811, 107° 34.159 |
|  | SCNA5c | orange | H01 | KX100167 | 24° 54.811, 107° 34.159 |
|  | SCMA2a | mandarin | H01 | KX100168 | 24° 54.761, 107° 34.111 |
|  | SCMA2b | mandarin | H03 | KX100169 | 24° 54.761, 107° 34.111 |
|  | SCMA5a | mandarin | H01 | KX100170 | 24° 54.767, 107° 34.073 |
|  | SCMA5b | mandarin | H01 | KX100171 | 24° 54.767, 107° 34.073 |
|  | SCLA2a | lime | H01 | KX100172 | 24° 54.698, 107° 33.994 |
|  | SCLA3a | lime | H01 | KX100173 | 24° 54.697, 107° 33.878 |
|  | SCLA3b | lime | H01 | KX100174 | 24° 54.697, 107° 33.878 |
|  | SCLA4a | lime | H01 | KX100175 | 24° 54.730, 107° 33.881 |
|  | SCTA4a | grapefruit | H01 | KX100176 | 24° 54.863, 107° 33.951 |
|  | SCTA5a | grapefruit | H01 | KX100177 | 24° 54.862, 107° 33.955 |
| Guasave, SL | SGNA4a | orange | H01 | KX100178 | 25° 38.452, 108° 31.512 |
|  | SGMA5a | mandarin | H01 | KX100179 | 25° 38.420, 108° 31.522 |
|  | SGLA1a | lime | H01 | KX100180 | 25° 06.150, 108° 01.580 |
|  | SGLA3a | lime | H01 | KX100181 | 25° 06.153, 108° 01.572 |
|  | SGLA5a | lime | H01 | KX100182 | 25° 06.157, 108° 01.557 |
|  | SGLA5b | Lime | H01 | KX100183 | 25° 06.157, 108° 01.557 |
| Mugica, MN | MMMA1b | mandarin | H04 | KX100184 | 18° 57' 51.0” 102° 10' 20.1'' |
|  | MMMA1c | mandarin | H04 | KX100185 | 18° 57' 51.0'', 102° 10' 20.1'' |
|  | MMMA1d | mandarin | H04 | KX100186 | 18° 57' 51.0'', 102° 10' 20.1'' |
|  | MMMA2a | mandarin | H04 | KX100187 | 18° 57' 51.0'', 102° 10' 23.5'' |
|  | MMMA2b | mandarin | H04 | KX100188 | 18° 57' 51.0'', 102° 10' 23.5'' |
|  | MMMA2c | mandarin | H04 | KX100189 | 18° 57' 51.0'', 102° 10' 23.5'' |
|  | MMMA2d | mandarin | H04 | KX100190 | 18° 57' 51.0'', 102° 10' 23.5'' |
|  | MMMA3d | mandarin | H04 | KX100191 | 18° 57' 49.5'', 102° 10' 21.1'' |
|  | MMMA3e | mandarin | H04 | KX100192 | 18° 57' 49.5'', 102° 10' 21.1'' |
|  | MMMA4a1 | mandarin | H04 | KX100193 | 18° 57' 48.7'', 102° 10' 20.1'' |
|  | MMMA4a2 | mandarin | H04 | KX100194 | 18° 57' 48.7'', 102° 10' 20.1'' |
|  | MMMA4b | mandarin | H04 | KX100195 | 18° 57' 48.7'', 102° 10' 20.1'' |
|  | MMMA4c | mandarin | H05 | KX100196 | 18° 57' 48.7'', 102° 10' 20.1'' |
|  | MMMA5a | mandarin | H04 | KX100197 | 18° 57' 48.9'', 102° 10' 21.2'' |
|  | MMTA1a | grapefruit | H01 | KX100198 | 18° 58' 0.50'', 102° 10' 16.9'' |
|  | MMTA2a | grapefruit | H04 | KX100199 | 18° 58' 08.3'', 102° 10' 16.7'' |
|  | MMTA2b | grapefruit | H04 | KX100200 | 18° 58' 08.3'', 102° 10' 16.7'' |
|  | MMTA2c | grapefruit | H04 | KX100201 | 18° 58' 08.3'', 102° 10' 16.7'' |
|  | MMTA3a | grapefruit | H04 | KX100202 | 18° 58' 08.6'', 102° 10' 15.3'' |
|  | MMTA3b | grapefruit | H04 | KX100203 | 18° 58' 08.6'', 102° 10' 15.3'' |
|  | MMTA3d | grapefruit | H04 | KX100204 | 18° 58' 08.6'', 102° 10' 15.3'' |
|  | MMTA4a | grapefruit | H04 | KX100205 | 18° 58' 07.4'', 102° 10' 15.4'' |
|  | MMTA4b | grapefruit | H01 | KX100206 | 18° 58' 07.4'', 102° 10' 15.4'' |
|  | MMTA4d | grapefruit | H04 | KX100207 | 18° 58' 07.4'', 102° 10' 15.4'' |
|  | MMTA4e | grapefruit | H04 | KX100208 | 18° 58' 07.4'', 102° 10' 15.4'' |
|  | MMTA5a | grapefruit | H01 | KX100209 | 18° 58' 09.2'', 102° 10' 16.2'' |
|  | MMTA5d | grapefruit | H06 | KX100210 | 18° 58' 09.2'', 102° 10' 16.2'' |
|  | MMTA5e | grapefruit | H04 | KX100211 | 18° 58' 09.2'', 102° 10' 16.2'' |
|  | MMLA1a | lime | H01 | KX100212 | 18° 57' 16.4'', 102° 08' 15.1'' |
|  | MMLA1b | lime | H04 | KX100213 | 18° 57' 16.4'', 102° 08' 15.1'' |
|  | MMLA1c | lime | H04 | KX100214 | 18° 57' 16.4'', 102° 08' 15.1'' |
|  | MMLA2a | lime | H04 | KX100215 | 18° 57' 16.1'', 102° 08' 11.3'' |
|  | MMLA3c | lime | H07 | KX100216 | 18° 57' 16.1'', 102° 08' 11.3'' |
|  | MMLA4c1 | lime | H08 | KX100217 | 18° 57' 15.6'', 102° 08' 12.1'' |
|  | MMLA4C2 | lime | H04 | KX100218 | 18° 57' 15.6'', 102° 08' 12.1' |
|  | MMNA1a | orange | H09 | KX100219 | 18° 58' 21.1'', 102° 04' 50.0'' |
|  | MMNA2a | orange | H01 | KX100220 | 18° 58' 20.5'', 102° 04' 49.5'' |
|  | MMNA3a | orange | H01 | KX100221 | 18° 58' 20.0'', 102° 04' 49.9'' |
|  | MMNA3c | orange | H01 | KX100222 | 18° 58' 20.0'', 102° 04' 49.9'' |
|  | MMNA4a | orange | H04 | KX100223 | 18° 58' 20.1'', 102° 04' 50.0'' |
|  | MMNA4c | orange | H01 | KX100224 | 18° 58' 20.1'', 102° 04' 50.0'' |
|  | MMNA4d | orange | H04 | KX100225 | 18° 58' 20.1'', 102° 04' 50.0'' |
|  | MMNA5b | orange | H01 | KX100226 | 18° 58' 20.0'', 102° 04' 49.8'' |
| Martínez de la Torre, VZ | VMNA1b | orange | H04 | KX100227 | 20° 12' 14.8'', 097° 04' 55.5'' |
|  | VMNA2b | orange | H04 | KX100228 | 20° 07' 14.2'', 097° 02' 54.9'' |
|  | VMNA5b | orange | H01 | KX100229 | 20° 07' 14.7'', 097° 02' 55.0'' |
|  | VMLA1c | lime | H10 | KX100230 | 20° 07' 46.7'', 097° 02' 56.7'' |
|  | VMLA1d | lime | H01 | KX100231 | 20° 07' 46.7'', 097° 02' 56.7'' |
|  | VMLA2b | lime | H04 | KX100232 | 20° 07' 44.8'', 097° 02' 56.4'' |
|  | VMLA3a | lime | H04 | KX100233 | 20° 07' 39.5'', 097° 02' 57.3'' |
|  | VMLA3b | lime | H04 | KX100234 | 20° 07' 39.5'', 097° 02' 57.3'' |
|  | VMLA4b | lime | H01 | KX100235 | 20° 07' 38.5'', 097° 02' 56.5'' |
|  | VMLA4c | lime | H10 | KX100236 | 20° 07' 38.5'', 097° 02' 56.5'' |
|  | VMLA4d | lime | H10 | KX100237 | 20° 07' 38.5'', 097° 02' 56.5'' |
|  | VMLA4e | lime | H01 | KX100238 | 20° 07' 38.5'', 097° 02' 56.5'' |
|  | VMLA5a | lime | H10 | KX100239 | 20° 07' 45.3'', 097° 02' 56.9'' |
|  | VMLA5b | lime | H04 | KX100240 | 20° 07' 45.3'', 097° 02' 56.9'' |
|  | VMLA5c | lime | H01 | KX100241 | 20° 07' 45.3'', 097° 02' 56.9'' |
|  | VMLA5d | lime | H04 | KX100242 | 20° 07' 45.3'', 097° 02' 56.9'' |
|  | VMLA5e | lime | H04 | KX100243 | 20° 07' 45.3'', 097° 02' 56.9'' |
|  | VMMA2a | mandarin | H04 | KX100244 | 20° 07' 41.0'', 097° 03' 03.3'' |
|  | VMMA2b | mandarin | H04 | KX100245 | 20° 07' 41.0'', 097° 03' 03.3'' |
|  | VMMA2c | mandarin | H01 | KX100246 | 20° 07' 41.0'', 097° 03' 03.3'' |
|  | VMMA2d | mandarin | H01 | KX100247 | 20° 07' 41.0'', 097° 03' 03.3'' |
|  | VMMA2e | mandarin | H01 | KX100248 | 20° 07' 41.0'', 097° 03' 03.3'' |
|  | VMMA3a | mandarin | H01 | KX100249 | 20° 07' 40.3'', 097° 03' 02.5'' |
|  | VMMA4a | mandarin | H04 | KX100250 | 20° 07' 39.1'', 097° 03' 02.5'' |
|  | VMMA4b | mandarin | H04 | KX100251 | 20° 07' 39.1'', 097° 03' 02.5'' |
|  | VMMA4c | mandarin | H01 | KX100252 | 20° 07' 39.1'', 097° 03' 02.5'' |
|  | VMMA4d | mandarin | H01 | KX100253 | 20° 07' 39.1'', 097° 03' 02.5'' |
|  | VMMA4e | mandarin | H01 | KX100254 | 20° 07' 39.1'', 097° 03' 02.5'' |
|  | VMMA5b | mandarin | H01 | KX100255 | 20° 07' 39.6'', 097° 03' 05.7'' |
| Montemorelos, NL | NLMMA1 | mandarin | H10 | KX100256 | 25° 15' 20.1'', 99° 46' 16.2'' |
|  | NLMMA3a | mandarin | H10 | KX100257 | 25° 15' 20.2'', 99° 46' 16.8'' |
|  | NLMMA3b | mandarin | H10 | KX100258 | 25° 15' 20.2'', 99° 46' 16.8'' |
|  | NLMMA3c | mandarin | H10 | KX100259 | 25° 15' 20.2'', 99° 46' 16.8'' |
|  | NLMNA3e | orange | H10 | KX100260 | 25° 15' 21.2'', 99° 46' 17.1'' |
|  | NLMNA5b | orange | H10 | KX100261 | 25° 15' 22.3'', 99° 46' 15.0'' |
|  | NLMNA5d | orange | H10 | KX100262 | 25° 15' 22.3'', 99° 46' 15.0'' |
|  | NLMTA4b | grapefruit | H10 | KX100263 | 25° 15' 31.9'', 99° 46' 24.3'' |
|  | NLMLA1a | lime | H10 | KX100264 | 25° 15' 19.9'', 99° 46' 14.0'' |
| Oxkutzcab, YN | YOTA1a | grapefruit | H11 | KX100265 | 20° 11' 27.6'', 89° 30' 36.5'' |
|  | YOTA1b | grapefruit | H04 | KX100266 | 20° 11' 27.6'', 89° 30' 36.5'' |
|  | YOTA2a | grapefruit | H04 | KX100267 | 20° 11' 28.0'', 89° 30' 37.3'' |
|  | YOTA2b | grapefruit | H04 | KX100268 | 20° 11' 28.0'', 89° 30' 37.3'' |
|  | YOTA3a | grapefruit | H04 | KX100269 | 20° 11' 28.5'', 89° 30' 37.3'' |
|  | YOTA3b | grapefruit | H04 | KX100270 | 20° 11' 28.5'', 89° 30' 37.3'' |
|  | YOTA4a | grapefruit | H04 | KX100271 | 20° 11' 28.5'', 89° 30' 37.3'' |
|  | YOTA4b | grapefruit | H04 | KX100272 | 20° 11' 28.5'', 89° 30' 37.3'' |
|  | YOTA4c | grapefruit | H04 | KX100273 | 20° 11' 28.5'', 89° 30' 37.3'' |
|  | YOTA4d | grapefruit | H04 | KX100274 | 20° 11' 28.5'', 89° 30' 37.3'' |
|  | YOTA5a | grapefruit | H04 | KX100275 | 20° 11' 28.1'', 89° 30' 37.7'' |
|  | YONA1a1 | orange | H04 | KX100276 | 20° 11' 16.6'', 89° 30' 54.0'' |
|  | YONA1a2 | orange | H04 | KX100277 | 20° 11' 16.6'', 89° 30' 54.0'' |
|  | YONA3b1 | orange | H04 | KX100278 | 20° 11' 17.0'', 89° 30' 53.6'' |
|  | YONA3b2 | orange | H04 | KX100279 | 20° 11' 17.0'', 89° 30' 53.6'' |
|  | YONA4a2 | orange | H04 | KX100280 | 20° 11' 16.9'', 89° 30' 52.5'' |
|  | YONA5b | orange | H04 | KX100281 | 20° 11' 17.4'', 89° 30' 53.9'' |
|  | YOLA1a | lime | H01 | KX100282 | 20° 11' 16.7'', 89° 30' 54.6'' |
|  | YOLA1c | lime | H04 | KX100283 | 20° 11' 16.7'', 89° 30' 54.6'' |
|  | YOLA3a1 | lime | H01 | KX100284 | 20° 11' 18.6'', 89° 30' 54.9'' |
|  | YOLA3a2 | lime | H01 | KX100285 | 20° 11' 18.6'', 89° 30' 54.9'' |
|  | YOLA3b2 | lime | H04 | KX100286 | 20° 11' 18.6'', 89° 30' 54.9'' |
|  | YOMA1a | mandarin | H04 | KX100287 | 20° 11' 17.1'', 89° 31' 24.7'' |
|  | YOMA1b | mandarin | H04 | KX100288 | 20° 11' 17.1'', 89° 31' 24.7'' |
|  | YOMA1c | mandarin | H04 | KX100289 | 20° 11' 17.1'', 89° 31' 24.7'' |
|  | YOMA2c | mandarin | H04 | KX100290 | 20° 11' 17.8'', 89° 31' 24.4'' |
|  | YOMA4a | mandarin | H04 | KX100291 | 20° 23' 57.4'', 89° 26' 45.6'' |
|  | YOMA4b | mandarin | H04 | KX100292 | 20° 23' 57.4'', 89° 26' 45.6'' |
