# Supplementary material for: Analysis of Genetic Variation in Brevipalpus yothersi (Acari: Tenuipalpidae) Populations from Four Species of Citrus Host Plants
Source: PLoS One. 2016 Oct 13;11(10):e0164552. doi: 10.1371/journal.pone.0164552 (PMC5063392; doi:10.1371/journal.pone.0164552)
Supplement: S2 Table — GenBank accession numbers, locality and haplotype assignment after the haplotype network analysis are provided. List of sequences used as a reference for the genetic comparison are shown. VZ = Veracruz, NL = Nuevo Leon, YN = Yucatan. (DOC) [file pone.0164552.s002.doc]

**S2 Table. List of samples of *Brevipalpus californicus* obtained from different host plant species**. GenBank accession numbers, locality and haplotype assignment after the haplotype network analysis are provided. List of sequences used as a reference for the genetic comparison are shown. VZ=Veracruz, NL= Nuevo Leon, YN=Yucatan.

| Location | Sample | Host | Haplotype | GenBank | Geographical coordinates (North, West) |
| --- | --- | --- | --- | --- | --- |
| Oxkutzcab, YN | YOMA5b1 | mandarin | H1 | KX100293 | 20° 23' 56.8'', 89° 26' 45.7'' |
| Martinez de la Torre, VZ | VMNA1c2 | orange | H2 | KX100294 | 20° 12' 14.8'', 097° 04' 55.5'' |
|  | VMNA2a | orange | H2 | KX100295 | 20° 07' 14.2'', 097° 02' 54.9'' |
|  | VMLA4a | lime | H2 | KX100296 | 20° 07' 38.5'', 097° 02' 56.5'' |
|  | VMLA4f | lime | H2 | KX100297 | 20° 07' 38.5'', 097° 02' 56.5'' |
|  | VMMA5c | mandarin | H2 | KX100298 | 20° 07' 39.6'', 097° 03' 05.7'' |
| Montemorelos, NL | NLMMA1b | mandarin | H2 | KX100299 | 25° 15' 20.1'', 99° 46' 16.2'' |
|  | NLMMA2 | mandarin | H2 | KX100300 | 25° 15' 21.1'', 99° 46' 16.8'' |
|  | NLMMA2e | mandarin | H2 | KX100301 | 25° 15' 21.1'', 99° 46' 16.8'' |
|  | NLMMA4d | mandarin | H2 | KX100302 | 25° 15' 22.1'', 99° 46' 17.8'' |
|  | NLMMA4g | mandarin | H2 | KX100303 | 25° 15' 22.1'', 99° 46' 17.8'' |
|  | NLMMA5a | mandarin | H2 | KX100304 | 25° 15' 21.4'', 99° 46' 17.8'' |
|  | NLMMA1d | mandarin | H3 | KX100305 | 25° 15' 20.1'', 99° 46' 16.2'' |
|  | NLMNA1d | orange | H2 | KX100306 | 25° 15' 20.2'', 99° 46' 16.4'' |
|  | NLMNA2d | orange | H2 | KX100307 | 25° 15' 20.7'', 99° 46' 15.4'' |
|  | NLMNA2f | orange | H2 | KX100308 | 25° 15' 20.7'', 99° 46' 15.4'' |
|  | NLMNA3a | orange | H2 | KX100309 | 25° 15' 21.2'', 99° 46' 17.1'' |
|  | NLMNA4a | orange | H2 | KX100310 | 25° 15' 21.5'', 99° 46' 14.0'' |
|  | NLMNA4d | orange | H2 | KX100311 | 25° 15' 21.5'', 99° 46' 14.0'' |
|  | NLMNA4c | orange | H2 | KX100312 | 25° 15' 21.5'', 99° 46' 14.0'' |
|  | NLMNA4e | orange | H2 | KX100313 | 25° 15' 21.5'', 99° 46' 14.0'' |
|  | NLMNA5 | orange | H2 | KX100314 | 25° 15' 22.3'', 99° 46' 15.0'' |
|  | NLMNA5e | orange | H2 | KX100315 | 25° 15' 22.3'', 99° 46' 15.0'' |
|  | NLMNA5a | orange | H2 | KX100316 | 25° 15' 22.3'', 99° 46' 15.0'' |
|  | NLMLA6f1 | lime | H2 | KX100317 | 25° 15' 23.5'', 99° 46' 15.1'' |
|  | NLMLA6f2 | lime | H3 | KX100318 | 25° 15' 23.5'', 99° 46' 15.1'' |
|  | NLMTA2a | grapefruit | H2 | KX100319 | 25° 15' 30.3'', 99° 46' 24.5'' |
|  | NLMTA3f | grapefruit | H2 | KX100320 | 25° 15' 31.7'', 99° 46' 25.6'' |
|  | NLMTA5c | grapefruit | H2 | KX100321 | 25° 15' 31.3'', 99° 46' 23.0'' |
